# “It shouldn't be just hush‐hush”: A qualitative community‐based study of menstrual health communication among women in Philadelphia

**DOI:** 10.1111/psrh.12277

**Published:** 2024-07-29

**Authors:** Allison R. Casola, Lynette Medley, Brianna C. Kunes, Nya McGlone, Alexis Silverio

**Affiliations:** ^1^ Department of Family and Community Medicine Sidney Kimmel Medical College, Thomas Jefferson University Philadelphia Pennsylvania USA; ^2^ No More Secrets Mind Body Spirit Inc. Philadelphia Pennsylvania USA; ^3^ Sidney Kimmel Medical College Thomas Jefferson University Philadelphia Pennsylvania USA

**Keywords:** communication, community health, menstrual health, menstruation, norms

## Abstract

**Introduction:**

Although menstruation is a natural biological process, many people feel embarrassed of their menses and struggle to discuss it. To mitigate menstrual communication stigma, it is necessary to first elucidate communication experiences and perceptions. Thus, we qualitatively explore menstrual communication among cisgender women who menstruate and their family, friends, healthcare providers, and community.

**Methodology:**

In partnership with No More Secrets (NMS), a Philadelphia menstrual health non‐profit, we conducted a community‐based participatory research (CBPR) project in Fall 2020. Cisgender, menstruating individuals ages 18–45 recruited from NMS' catchment in Philadelphia participated in semi‐structured interviews about their menstrual experiences and communication (*N* = 20). A deductive, theory‐driven approached based on the social‐ecological model was used to analyze the data.

**Results:**

Varying emotional responses arose across social‐ecological levels: communication was awkward and simplistic with family; positive and supportive with friends and community members; and uncomfortable and frustrating with healthcare providers. Participants echoed the importance of menstrual communication as a means of sharing information, feeling less alone, and decreasing menstrual stigma.

**Discussion:**

Findings can inform future CBPR workshops that address stigma in familial, healthcare, and community‐based discussions to improve menstrual health and experiences for cisgender girls and women, transgender men, and gender non‐binary individuals who menstruate.

## INTRODUCTION

Menstruation is a natural physiological process. On a given day, 800 million people are menstruating worldwide, and across the lifespan a person who menstruates can expect to have over 400 menstrual periods.^1^ Although menstruation is normal, it remains a highly stigmatized topic.^1^, ^2^, ^3^ Menstrual stigma leaves many feeling ashamed and embarrassed about their menses and menstrual experiences.^2^, ^3^ These feelings, in combination with societal menstrual taboos, can discourage menstrual communication between menstruators and their family, friends, and healthcare professionals.^3^

The consequences of silence around menstruation are profound and persistent. Silence allows for the perpetuation of misinformation and a general misunderstanding of what constitutes a physiologically normal menstrual cycle, leaving many young people unprepared for menarche.^4^, ^5^ This limited understanding also leads to underutilization of reproductive health services, like gynecological exams and pap smears, and exacerbates undue suffering from untreated disease.^6^ Additionally, limited menstrual communication exacerbates feelings of isolation and fear regarding menses^4^, ^5^ and maintains inaccessibility to menstrual resources and products.^7^ These harmful effects of menstrual stigma are generationally perpetuated, reinforcing unhealthy norms and a spiral of menstrual silence.^3^

Open and supportive communication about menstrual experiences and attitudes could help diminish menstrual stigma and its consequences.^3^ However, little research exploring current menstrual communication norms exists,^3^, ^8^ and work at the community level is particularly lacking. Much of the previous literature regarding menstrual health communication norms is dated, with many studies well over a decade old at the time of data collection for the present work (fall, 2020).^5^, ^9^, ^10^, ^11^ Additionally, previous research tends to focus on adolescent health^11^, ^12^ and menstrual health knowledge, practices, and attitudes,^8^ usually in an international setting.^12^, ^13^ As a result, researchers have little understanding of the current values and needs of cisgender women, transgender men, and gender non‐binary individuals who menstruate domestically^13^ and cannot sensitively, knowledgably, and efficaciously encourage positive communication to destigmatize menstruation (Table 1).

**TABLE 1:** Menstrual communication across ecological groups and perceived communication importance, with corresponding exemplar quotes from the text.

Ecological level	Exemplar quote
Family	“That's something that my mother didn't go over with us. I don't know. I don't know if she was so busy or if maybe she felt a little uncomfortable, but I think at first, I did feel uncomfortable because I guess it's one of those things, like once you start talking about the menstrual cycle, you have to talk about, and of course, because you're explaining to your young child or this young woman that this is the time where your egg is released and you got to kind of get into it. Like you got to kind of get into the birds and the bees. So, maybe she didn't feel comfortable talking about it. So, maybe that was why… I don't know.” “I was young and I was scared of my mom, not scared but I didn't want my mom to know certain things.” “I also have little sister, told her like ‘You have to find whatever's comfortable for you,’ about pads sizes and how you are wearing them, make sure you have the proper pad on, and make sure that it's comfortable for you, that it's okay if you get it on your clothes.”
Friends and community	“Certain conversations would be how we would make our own pads when we didn't have any and ways that we got creative with doing so, trying to get pain medication for being in pain, things of that sort.” “I would say, when I talked to my friends about it … we give tips and we could share experience. I mentioned earlier, how I would think that I will have more of a lighter period and some of my friends they will have lighter or more irregular periods. Not more so comparing, but just sharing our experiences together.” [No More Secrets would say] “I can't tell you about your body, but maybe you should try this out, maybe you should try the cup, maybe you should try the tampon, maybe. And, you know what? If that don't work, let me know, come back and I'll give you this.”
Healthcare providers	“I kind of felt that conversation I know they didn't even care, like she knew it was just, I guess, it was just procedure, the questions she had to ask me. But you could tell it wasn't like a genuine connection. Whereas though she wanted to be there doing that. It was just like a bad advice.” “Made me feel unheard because this wasn't my first time trying that. I went to the doctor, I don't even like going to the doctors, but I went there about four times for them to give me the same response after I told them the three remaining other times that it does not work. So now it's just, y'all just prescribing me these pills to get me out y'all face, just shutting me up and it ain't going [away].” “They may ask the question like, “When was the last date of your period,” but besides that, no.”

	Themes	Exemplar quote
Communication: Promotion of menstrual empowerment	Management	“Because I may be going through something with my periods and I need somebody else who's already gone through that same thing, that same issue that I'm dealing with right now when it came to their period, but they may have been able to overcome that, get help and you know, each way something. So I, it give me an idea of the steps that I should take to go about my situation that I have with my cycle.” “Because once you talk about it, you can learn certain things to do to help it get better. So for example, if you are talking about how you're having one and it's not going so good. If you're on birth control, then that could be the reason why it's not going so good, because you can have a certain kind of birth control, which makes it more heavy. So once you get off and you can change the method of birth control, if possible, it might make it more lighter. And that could make it better for the future.”
	Feel less alone	“I think that that's really important to be able to communicate and work through those different emotions and those feelings just so everybody can understand; you become a little bit more sensitive to other people's needs; that at first, it's uncomfortable and it may be a little awkward, but… eventually better.” “I guess growing up, you just think of your period as something private and that might hurt you in the long run because you're not talking about it. People don't know. You could help younger people learn what to expect. I guess it's such a touchy situation. I don't know. I mean, it's a great situation, but it's womanhood. It helps you grow up.”
	Decrease menstrual stigma	“Talking about your period is definitely kind of a taboo topic, even though as a woman, we all have periods. It's funny because a lot of us will use a different terminology when we want to express the fact that we're on our periods. Which is perfectly fine because it makes you uncomfortable but when we use terminology like, ‘It's my cycle’ or ‘my time of the month’ or ‘my girl was in town’ or something like that. I think if we normalize talking about it more, we wouldn't feel… A lot of women wouldn't feel as embarrassed about it.” “I just think that women should be able to talk about periods in a regular conversation without people looking in disgust because it's just something natural.”

This project aims to address these gaps in knowledge using community‐based participatory research to explore the menstrual health experiences of cisgender women, ages 18–45, in Philadelphia, PA. Through qualitative interviewing, we sought to conceptualize menstrual health communication across social‐ecological levels with friends, family, healthcare providers, and community members. Further, we define the feelings associated with these conversations and the perceived importance of menstrual communication. For this study, menstrual communication refers to conversation regarding any aspect of menstruation including but not limited to menarche, cycle characteristics, and obtaining and using hygiene products. Findings from this work can inform community workshop development and educational programs related to menstrual health that aim to destigmatize menstrual communication.

## METHODS

### CBPR academic‐community partnership

No More Secrets Mind Body Spirit, Inc. (NMS) is a grassroots sexuality awareness organization that has developed the only menstrual product bank and in‐home delivery service in Philadelphia and the only brick‐and‐mortar Menstrual Hub and Uterine Care Community Center in the United States.^14^ NMS works solely off donations to spread awareness about period poverty, advocate for menstrual equity, and deliver bags filled with 3 months' worth of hygiene products to people in need.^14^ Their catchment population tends to be individuals living in Philadelphia's most poverty‐stricken neighborhoods, particularly in North and West Philadelphia.

### Design and sample

Study authors connected at a community‐driven research event in Philadelphia, PA (December 2019). The event introduces academic researchers and community partners with common research goals to facilitate the formation of community‐based participatory research (CBPR) teams to advance the mission of community agencies.^15^ From this meeting, the academic and community partners formed a CBPR team to conceptualize, develop, and implement this study.^16^

Participants were recruited from NMS's Philadelphia catchment population using convenience sampling (*N* = 20). Eligible participants included cisgender women, ages 18–45, who received a menstrual hygiene bag from NMS in the last 2 years. These individuals received an IRB‐approved recruitment letter or phone call during which a member of the research team read the recruitment letter aloud. The authors recognize not all people that identify as women menstruate and not all people who menstruate identify as women. However, this study specifically recruited cisgender women who menstruate to avoid confounders associated with the intersectionality of social vulnerabilities and inequities of multiple‐marginalized groups within the small sample size.

### Data collection procedures and interview guide

Interviews occurred in Fall 2020 via telephone by having each participant call into an audio‐recorded Zoom meeting. Members of the research team took turns conducting the interviews following a flexible, semi‐structured guide of pre‐determined questions and question probes (Appendix A). NMS' prior work in the community informed the development of the interview guide, which was pilot‐tested and revised prior to data collection.

To protect anonymity, information collected from each participant received a unique identifier. Participants received a $5 Walmart gift card and a three‐month menstrual supply bag upon completion of the interview. Interview questions asked about general menstrual health experiences, menstrual communication among friends, family, community members, and healthcare providers, and how communication could impact menstrual experiences. The Thomas Jefferson University Institutional Review Board (#20G.743) approved this study, and all participants consented to the interview and audio recording process.

### Data analysis

A professional transcriptionist transcribed interview recordings. A deductive, theory‐driven coding approach was applied, using pre‐determined codes and fitting the data as appropriate. The social‐ecological model, a well‐known theoretical framework in health behavior and promotion, was used to guide this work. The theory postulates that there are multiple levels of influence that can impact health, ranging from intrapersonal factors, interpersonal factors, and societal/environmental factors.^17^ These factors change with time, directly influencing individual's development and beliefs over their lifespan.^17^ The social ecological model highlights the complexity of one's actions within their environment.^17^ As menstrual health in itself is complex and multilayered, it is well suited for examination under a social‐ecological lens.^3^, ^18^ To explore menstrual communication across the social ecosystems, our codebook was developed a priori based on the social‐ecological model levels: communication with family, friends, healthcare providers, and community agencies/members. Data were then sorted into organizational categories based on communication at the aforementioned levels.

However, an inductive approach was used to analyze the question “Do you think talking about your period could lead to better menstrual experiences?” surrounding the importance of menstrual communication. Specifically, the Key Words in Context approach, presented by Ryan and Bernard,^19^ that employs a three‐level approach to ensure consensus and to maximize the generation of ideas.^20^ Beginning with Level I, transcripts were read and re‐read to identify keywords to develop preliminary codes. Level II coding ascertained associated patterns presented in the text from Level I, and Level III coding organized the identified patterns in Level II into themes.^20^

## RESULTS

Interviews lasted 07:49–36:21 min with an average length of 17:16 min, resulting in 149 pages of transcripts. The average age of participants (*N* = 20) was 20 years (SD = 8.9) and 17 (85%) were Black or African American. Nearly two‐thirds of participants reported some college education but no degree (65%, *n* = 13); with 50% currently living in a North Philadelphia zip code. Mean age of menarche was 12 years (SD = 2.1).

**FIGURE 1:**
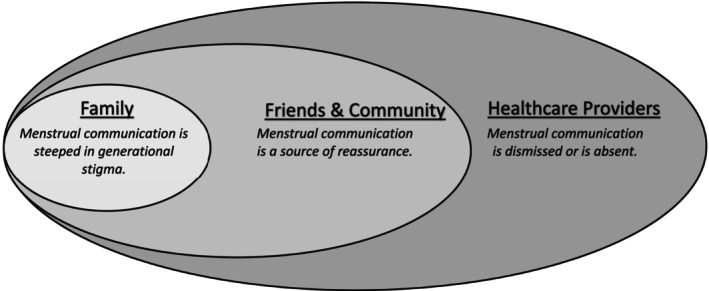
Modified social‐ecological model depicting themes related to menstrual communication.

Qualitative findings are descriptively listed by social‐ecological groups (Figure 1) and by perceived communication importance.

### Family: Menstrual communication steeped in generational stigma

Participants reported talking about their menses to a family member, most often their mother, followed by sister, daughter, grandmother, aunt, cousin, and niece, respectively. The initial conversations that most participants had about menses were largely restricted to general menstrual symptoms: “I just tell them it's on. My period is on, it's kind of heavy, so I'm going to be sitting down some” and symptom management like over‐the counter medication and heating pads, pads and tampons, and resources to access products. The limited conversation with their mothers and family led a small number to feel scared, overwhelmed, confused, or uncomfortable about menstruation, as one stated their mother only said, “make sure that you're cleaning yourself and make sure that you fold it [pad] up in a way, and make sure that no one can tell”: highlighting generational stigma around menstruation and puberty conversations. Another stated: “That's something that my mother didn't go over with us. I don't know. I don't know if she was so busy or if maybe she felt a little uncomfortable, but I think at first, I did feel uncomfortable because I guess it's one of those things, like once you start talking about the menstrual cycle, you have to talk about, and of course, because you're explaining to your young child or this young woman that this is the time where your egg is released and you got to kind of get into it. Like you got to kind of get into the birds and the bees. So, maybe she didn't feel comfortable talking about it. So, maybe that was why… I don't know.”

However, a few have noted that they are using these communication experiences to support others who are menstruating, noting they now feel proud to be there for their younger family members and future children: “I also have a little sister, told her like ‘You have to find whatever's comfortable for you,’ about pads sizes and how you are wearing them, make sure you have the proper pad on, and make sure that it's comfortable for you, that it's okay if you get it on your clothes.”

### Friends and community: Menstrual communication is a source of reassurance

Almost all participants talked to their friends about their menses, typically about their general experiences, cycle characteristics (flow, duration), and associated symptoms (pain, cramps, bloating, migraines). Although all participants had received a menstrual supply bag from NMS within the past 2 years, only a handful of participants specifically said they talked with a teacher or community organization about their menses.

Here, menstrual communication connected friends and community members and served as a source of reassurance. Participants noted how happy they felt when they could relate to their friends and know they were not going through their menstrual concerns alone, commiserate about how messy their period can be, and general excitement for it to be over: “It makes me feel happy because I know that we can relate on something.” Others described that while conversations were still awkward at times, talking about menstruation with friends was comforting, with one participant describing it as a “sisterhood conversation” and another describing that their conversations with friends made them consider their options and products they use to manage their periods.

The participants who talked with community organization NMS discussed menstrual products and ways to access them, corresponding sizes to try, the realities of period poverty in the community, and general menstrual education, emphasizing that the organization showed them bodily respect and personal choice when it came to what works best for them individually during their period: [No More Secrets would say] “I can't tell you about your body, but maybe you should try this out, maybe you should try the cup, maybe you should try the tampon, maybe. And, you know what? If that don't work, let me know, come back and I'll give you this.”

### Healthcare providers: Menstrual communication is dismissed or absent

All participants mentioned talking to a healthcare provider, predominately about pain/cramps, cycle irregularity, heavy bleeding patterns, bloating, clotting, and pain management. Conversations were largely negative, and left participants feeling as though their concerns were going unheard and ultimately dismissed: “I kind of felt that conversation I know they didn't even care, like she knew it was just, I guess, it was just procedure, the questions she had to ask me. But you could tell it wasn't like a genuine connection.” Participants said they had to drive the conversations, some having to fully initiate the discussion themselves, and one mentioning that they felt so embarrassed and ashamed to bring up the topic, it took three visits before they could work up the courage to ask menstrual‐related questions. Hormonal contraception for the purpose of treating irregular menses was also a frequent topic. Although a few reported emotionally neutral discussions about hormonal contraception to regulate their cycle with their provider, others felt uncomfortable or forced, stating they were “put on birth control”, or that they “found out” they “had” to use hormonal contraception to regulate their cycle. Some participants emphasized how uncomfortable, impersonal, and disingenuous these experiences were, as doctors made them feel unheard and pushed hormonal contraception or other medications on them; even after repeatedly saying no: “prescribing me these pills to get me out y'all face, just shutting me up.” Lastly, a participant stated that although she felt fine with her female doctor, things were less comfortable once she got a male doctor, as he made her feel that her menstrual concerns were normal given her family history; another noted that the topic simply does not come up often.

### Communication: Promotion of menstrual empowerment

Participants shared their thoughts on the importance of menstrual communication, and it was clear they felt that discussing menstruation would lead to a feeling of empowerment. Specifically, participants described that menstrual communication can help people who menstruate to (1) improve menstrual management, (2) feel less alone, and (3) decrease overarching menstrual stigma.

#### Management

Several participants noted that talking about menstruation can lead to better menstrual management and experiences, through dialogue and information sharing of tips or ideas to improve cycle characteristics. They emphasized that if you do not discuss menstruation and advocate for yourself, you may never get help managing symptoms or understand what is happening inside your body: “once you talk about it, you can learn certain things to do to help it get better. So, for example, if you are talking about how you're having one and it's not going so good. If you're on birth control, then that could be the reason why it's not going so good, because you can have a certain kind of birth control, which makes it more heavy. So once you get off and you can change the method of birth control, if possible, it might make it more lighter. And that could make it better for the future.”

#### Feel less alone

Participants also noted that talking about menstruation can connect people who are going through the same thing, helping them feel less alone in their situation or experiences and empathizing with others: “I think that that's really important to be able to communicate and work through those different emotions and those feelings just so everybody can understand; you become a little bit more sensitive to other people's needs.” A few participants also stated that by talking about menstruation they could help connect with younger generations, helping them learn what to expect and explain that menstruation is not a bad thing: “People don't know. You could help younger people learn what to expect.”

#### Decrease menstrual stigma

Participants emphasized that it is important to talk about menstruation to decrease overall stigma around the topic, noting that it should not be as taboo as people make it: “it shouldn't be just as hush, hush…we should be able to talk about periods in a regular conversation without people looking in disgust because it's just something natural … [because] as a woman, we all have periods.” Others noted that talking will make people feel less uncomfortable, and help normalize the topic, saying that “It's funny because a lot of us will use a different terminology when we want to express the fact that we're on our periods…I think if we normalize talking about it more, we wouldn't feel… A lot of women wouldn't feel as embarrassed about it”.

## DISCUSSION

Despite its naturality, menstruation is a difficult topic for many people to discuss. Our qualitative analysis yielded rich insight into menstrual communication among women in Philadelphia, along with their perceived importance of communication. Menstrual communication generally covered participant's personal experiences, cycle characteristics, and symptom management. Across social‐ecological groups, menstrual‐related conversation was fact‐based and avoidant among family members, positive and supportive among friends and community members, and negative and frustrating with healthcare workers.

Menstrual communication among family members was most often focused on the facts—simple reproductive physiology, hygiene products, and systemic symptom management. The limited communication described between participants and their family members reflects previous findings that menstrual communication between mothers and daughters is largely awkward and negative.^5^, ^21^ This uneasy dialogue teaches young people with a uterus to embody avoidance as a means of social preservation^3^ and to adopt negative attitudes toward their own cycle, which they may subconsciously impress upon their future children, perpetuating a spiral of generational silence and stigma around menstruation.^4^ Notably, several participants admitted these uncomfortable conversations inspire them to be a knowledgeable resource for younger family members—casting light on their desire to break generational menstrual stigma.^5^ Educational programs focusing on intergenerational workshops and conversation starters could be useful for honing the confidence and communication skills necessary to minimize the spiral of menstrual silence among families.

Communication with friends and community members was unique compared to the other social‐ecological groups. Unlike with others, communication with friends and community made most participants happy and comforted to know they were not alone in their menstrual experiences. Notably, participants reported that menstrual communication with NMS centered around their individual needs, emphasizing the sisterhood atmosphere the organization fosters. The breadth of conversation with NMS was more variable than in any other category, covering all aspects of menstrual hygiene, period poverty, and individual experiences. The optimistic emotions associated with open menstrual conversation with friends and community reflects the importance of encouraging more supportive and positive communication in stressful situations like menstruation.^22^ Providing dedicated spaces similar to NMS for cisgender girls and women, transgender men, and gender non‐binary individuals who menstruate to safely discuss their experiences will continue to foster normalcy and security as menstrual communication norms. Additionally, increasing the number of settings where people feel comfortable talking about their cycle is beneficial to diminishing feelings of isolation, suffering, and embarrassment shared among all who menstruate.^22^

All participants discussed their cycle with a healthcare provider. Historically, women tend to feel embarrassed or afraid to seek medical care for menstrual health concerns^6^ for fear of mockery, dismissal, or judgment.^6^, ^23^ These feelings are especially common among persons of color who regularly experience implicit and overt racism in healthcare settings.^3^, ^24^ Unfortunately, our results demonstrate similar experiences. With healthcare providers, participants felt they needed to initiate or drive menstrual conversation, felt that the provider was impersonal or disingenuous, and described provider's emphasis on “pushing” hormonal contraception to manage menses. This finding suggests that primary care physicians are key to improving patient‐provider menstrual communication. Given their role as entry points into the healthcare system, expertise in providing care over the lifespan, and equitable, prevention‐based wellness approach to health,^25^ these providers can initiate and maintain consistent conversations about menstruation early in adolescence.^4^ This way, providers can build rapport with patients to normalize menstrual conversations from menarche to menopause.

Participants almost unanimously agreed that talking about menstruation was important and valued the benefits of open, supportive menstrual communication. These are encouraging findings and present opportunities for public health interventions. Participants asserted that improved menstrual communication can improve individual menstrual experiences, minimize feelings of loneliness, and mitigate menstrual stigma.^3^, ^4^, ^18^, ^22^ Health behavior theory underscores individual understanding and value placement as a precursor to behavioral change (i.e. promoting menstrual communication). However, the societal ecosystem could inhibit such progress. Therefore, public health researchers should work to dismantle cultural stigmatization of menstruation through the strategic use of social and behavioral change communication (SBCC). SBCC provides messaging and interactive activities for individuals and communities regarding a public health concern, such as menstrual communication, to educate and promote change in health behavior across the social‐ecological model.^26^ SBCC has previously demonstrated success in improving adolescent girls' reproductive health knowledge, attitudes toward gender, and menstrual communication.^27^ Thus, implementing SBCC projects that highlight menstruation's naturality and key application for reproduction may reduce stigma and normalize day‐to‐day menstrual communication.

### Strengths and limitations

The biggest strength of this study is its CBPR approach, allowing us to gain critical insight into the menstrual health communication patterns that persist across the social‐ecological landscape in a way that is respectful, collaborative, and wanted by community leaders. Data presented are from a larger qualitative study on menstrual health that explores menstrual communication, experiences, attitudes, and normative beliefs in the community, developed and implemented jointly by the collaborative team. We urge researchers to continue studying menstrual health topics in the United States using collaborative processes, such as CBPR, to fully elucidate its scope across social‐ecological levels.

Our study focused on cisgender women from Philadelphia who have utilized supports from a local menstrual health organization and employed a convenience sampling approach, thereby limiting geographic and demographic generalizability. However, we expect many of these communication patterns to exist among other urban cisgender women who menstruate in cities like Philadelphia. Given the qualitative nature of this work, the stigmatized topic, and the fact that data collection occurred in the summer/fall of 2020, coinciding with the COVID‐19 pandemic, Black Lives Matter protests, and 2020 general election, recall bias may be possible.

## CONCLUSION

While the present study finds discomfort persists related to menstrual communication across various social‐ecological levels, participants enthusiastically supported the need for and importance of improved menstrual communication. Further examination of communication pathways and strategies to foster supportive discussion channels is necessary, as menstrual communication and menstrual health, wellness, and dignity are inextricably linked.

## AUTHOR CONTRIBUTIONS

ARC was the academic co‐principal investigator and was involved with project conceptualization and development, conducting interviews, data coding and analysis, and report writing. LM was the community‐based co‐principal investigator involved with project conceptualization and development, conducting interviews, data coding and analysis, and report writing. BCK participated in project conceptualization and development, conducting interviews, data coding and analysis, and report writing. NM participated in project conceptualization and development, conducting interviews, data coding and analysis, and report writing. AS participated in conducting interviews, data coding and analysis, report writing, and general project management.

**MENSTRUAL HEALTH EXPERIENCES AND COMMUNICATION NORMS AMONG WOMEN IN PHILADELPHIA**

## APPENDIX A INTERVIEW GUIDE

**[So that is the end of my list of questions. Thank you for your time – we really appreciate it. I’m now going to turn off the recording and we can confirm your address for the gift card and supply bag.]**

**[TURN OFF RECORDING]**
